# The Aerobic and Cognitive Exercise Study (ACES) for Community-Dwelling Older Adults With or At-Risk for Mild Cognitive Impairment (MCI): Neuropsychological, Neurobiological and Neuroimaging Outcomes of a Randomized Clinical Trial

**DOI:** 10.3389/fnagi.2018.00076

**Published:** 2018-05-04

**Authors:** Cay Anderson-Hanley, Nicole M. Barcelos, Earl A. Zimmerman, Robert W. Gillen, Mina Dunnam, Brian D. Cohen, Vadim Yerokhin, Kenneth E. Miller, David J. Hayes, Paul J. Arciero, Molly Maloney, Arthur F. Kramer

**Affiliations:** ^1^The Healthy Aging and Neuropsychology Lab, Union College, Schenectady, NY, United States; ^2^Alzheimer's Disease Center, Albany Medical Center, Albany, NY, United States; ^3^Sunnyview Rehabilitation Hospital, Schenectady, NY, United States; ^4^Stratton VA Medical Center, Albany, NY, United States; ^5^Department of Biology, Union College, Schenectady, NY, United States; ^6^Biomedical Sciences Department, Oklahoma State University, Tulsa, OK, United States; ^7^Department of Anatomy and Cell Biology, Oklahoma State University, Tulsa, OK, United States; ^8^Department of Health & Human Physiological Sciences, Skidmore College, Saratoga Springs, NY, United States; ^9^Beckman Institute, University of Illinois, Urbana-Champaign, Champaign, IL, United States

**Keywords:** cognitive, exercise, aging, MCI, dementia, neuropsychological, exergame, Alzheimer's disease

## Abstract

Prior research has found that cognitive benefits of physical exercise and brain health in older adults may be enhanced when mental exercise is interactive simultaneously, as in exergaming. It is unclear whether the cognitive benefit can be maximized by increasing the degree of mental challenge during exercise. This randomized clinical trial (RCT), the Aerobic and Cognitive Exercise Study (ACES) sought to replicate and extend prior findings of added cognitive benefit from exergaming to those with or at risk for mild cognitive impairment (MCI). ACES compares the effects of 6 months of an *exer-tour* (virtual reality bike rides) with the effects of a more effortful *exer-score* (pedaling through a videogame to score points). Fourteen community-dwelling older adults meeting screening criteria for MCI (sMCI) were adherent to their assigned exercise for 6 months. The primary outcome was executive function, while secondary outcomes included memory and everyday cognitive function. Exer-tour and exer-score yielded significant moderate effects on executive function (Stroop A/C; *d*'s = 0.51 and 0.47); there was no significant interaction effect. However, after 3 months the exer-tour revealed a significant and moderate effect, while exer-score showed little impact, as did a game-only condition. Both exer-tour and exer-score conditions also resulted in significant improvements in verbal memory. Effects appear to generalize to self-reported everyday cognitive function. Pilot data, including salivary biomarkers and structural MRI, were gathered at baseline and 6 months; exercise dose was associated with increased BDNF as well as increased gray matter volume in the PFC and ACC. Improvement in memory was associated with an increase in the DLPFC. Improved executive function was associated with increased expression of exosomal miRNA-9. Interactive physical and cognitive exercise (both high and low mental challenge) yielded similarly significant cognitive benefit for adherent sMCI exercisers over 6 months. A larger RCT is needed to confirm these findings. Further innovation and clinical trial data are needed to develop accessible, yet engaging and effective interventions to combat cognitive decline for the growing MCI population.

ClinicalTrials.gov ID: NCT02237560

## Introduction

Annual diagnoses of dementia, such as due to Alzheimer's disease (AD), are expected to approach 1 million in 2050, more than double the new cases diagnosed in 2000 (Alzheimer's Association, 2014). Lacking a cure for any of the many causes of this devastating decline in cognitive function and loss of independence, a rising public health outcry has helped spark initiatives like the National Alzheimer's Project Act (2011) and the Healthy Brain Initiative (Alzheimer's Association and Centers for Disease Control Prevention, 2013). Research continues to press on, seeking innovative and empirically-validated ways to prevent or ameliorate cognitive decline and impairment.

While behavioral interventions are unlikely to completely prevent or halt dementia, there is the potential for physical exercise to reduce the risk of dementia onset (Heyn et al., 2004; Hillman et al., 2008) or slow progression (Larson, 2010). It has been estimated that if the onset of dementia could be delayed a few years, the impact on the population over time could be dramatic, decreasing prevalence by 1 million cases after 10 years in the United States alone. By also delaying progression for 2 years this could reduce dementia incidence by an astonishing 18 million cases globally per year (Sano et al., 1997; Brookmeyer et al., 1998). Interventions that reach patients before they have declined into diagnosable dementia can have the greatest impact. Thus, it is especially desirable to develop interventions that target those who may be at an intermediate stage, perhaps diagnosed with or at risk for mild cognitive impairment (MCI; per DSM-IV in 1994; Jak et al., 2009) also known more recently as mild neurocognitive disorder (mNCD per DSM-V in 2013; henceforth MCI will be used for simplicity; Roberts and Knopman, 2013; Jak et al., 2016).

### Neuropsychological effects of physical and cognitive exercise

Researchers have been investigating a variety of behavioral interventions, including exercise, to improve cognitive function and decrease risk of progression to dementia among individuals with MCI (e.g., Kramer et al., 1999; Colcombe and Kramer, 2003; Studenski et al., 2006; Angevaren et al., 2008; Baker et al., 2010; Geda et al., 2010; Martin et al., 2011). Physical exercise has garnered significant support in the literature for its potency in effecting cognitive benefits (e.g., Colcombe and Kramer, 2003). While the literature examining the relationship between physical exercise and cognitive benefit is not without issues (e.g., recent Cochrane Reviews; Forbes et al., 2015; Young et al., 2015, which focused on dementia and normative older adults, respectively), it is widely accepted that exercise benefits cognition, particularly executive function among symptomatic individuals, that are yet early in their cognitive decline (e.g., MCI), the very population on which this study focused (e.g., Colcombe and Kramer, 2003; Etnier and Chang, 2009; Geda et al., 2010; Gates, 2013; Hess et al., 2014; Zheng et al., 2016). Indeed, pre-clinical persons, ranging from asymptomatic older adults to those with early MCI, may experience the greatest benefit from exercise, due to the relatively preserved brain structures and functions (in contrast with more progressed dementia) that can support and supply components needed to realize neuroplasticity as triggered by exercise (e.g., Colcombe et al., 2004; Kramer and Erickson, 2007; Ahlskog et al., 2011; Teixeira et al., 2016). However, knowing that physical exercise may be good for one's body and brain may not be enough to induce regular practice of exercise. Most older adults do not meet the guideline from the American College of Sports Medicine (ACSM) which now recommends 45 min per day, 5–7 days per week, including vigorous exercise (Chodzko-Zajko et al., 2009).

**Figure 1:**
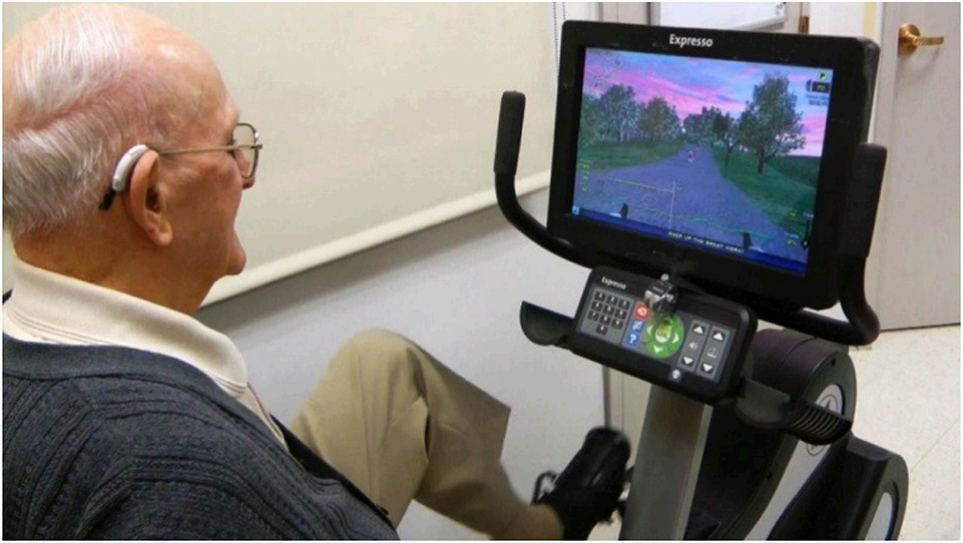
Stationary bike equipped with virtual reality display (aka “cybercycle” so named in our previous RCT; Anderson-Hanley et al., 2012). Former study participant demonstrating use of a cybercycle exergame; used with permission.

In a previous randomized clinical trial (Anderson-Hanley et al., 2012), we aimed to induce a thorough dose of exercise (Vidoni et al., 2015) by assigning older adults to an exergame^1^. We expected that the engagement of a stimulating exergame would motivate regular exercise, therefore allowing older adults to achieve maximum cognitive benefit. Three months of pedaling a virtual-reality-enhanced stationary ergometer, displaying scenic bike tours which we referred to as a “cybercycle” (see Figure 1), was compared with a traditional stationary bike. In the end, the cybercycle condition yielded significantly greater cognitive benefit, as hypothesized. However, surprisingly, it was not due to a difference in dose; we found instead that both the cybercycle and traditional cyclists has been similarly adherent (e.g., no significant difference between groups in miles, minutes, power, heart rate, etc.).

This unexpected finding raised the possibility that the screen display was providing benefit, beyond that of basic entertainment or motivation. We theorized the enhanced form of exercise was yielding findings much like those of the environmental enrichment literature (e.g., animals provided with greater physical and mental stimulation experience enhanced brain function; Pusic et al., 2016); thus it seems more (even two types at once) is generally better, although to a point. In humans the complexity of experience and the diversity of type, timing and compounding of “enrichment” makes this considerably challenging to study as a unique phenomenon. It seemed that for our participants, by interacting with the tour stimuli on screen (e.g., changing pedaling speed yielded a change in speed of scenery moving by), they were certainlyengaged in some additional amount of cognitive processing beyond the control condition. This layered cognitive stimulation may have combined with the physical exercise required of the exergame to create a “two-for-one” impact that resulted in the observed additional cognitive benefit for participants. Hence, we hypothesized that participants experienced better neuropsychological outcomes because of the potentially synergistic phenomena created by combining a type of “mental exercise” that was interactive with physical exercise (Anderson-Hanley et al., 2012; Frantzidis et al., 2014).

Several areas of the research literature speak to the possible validity of this hypothesized compounding or synergistic effect. As previously mentioned, there is a robust literature documenting the cognitive benefits of physical exercise. There is also a growing literature on cognitive training that purports significant effects (Willis et al., 2006; Karr et al., 2014; Toril et al., 2014; Wang et al., 2014; Huntley et al., 2015; Train the Brain Consortium, 2017). However, in our view, the claims are less convincing due to problems with reporting, limited generalizability, and other reasons that have been cited in various critiques of the literature and related “brain training” industry (e.g., Owen et al., 2010; Muijden et al., 2012; Redick et al., 2013; Boot and Kramer, 2014; Simons et al., 2016; Zokaei et al., 2016). Indeed, a Cochrane Review of 11 RCTs concluded there was “no indication of any significant benefits from cognitive training” (Bahar-Fuchs et al., 2013, p. 1). Nevertheless, considering some “mixed” results on the potential benefits of cognitive training, and as well as the animal literature on neurobiological impact of cognitive enrichments (reviewed below), there is potential for mental challenge to have its own benefit on cognition. Although perhaps it is most effectively implemented as part of a more naturalistic intervention, as when integrated with physical activity that taps additional neural mechanisms.

One way to examine this possibility further is to evaluate the body of literature that encompasses the cognitive effects of interventions with multiple components (e.g., cognitive training and physical exercise). Often these are referred to as:

- “combined” interventions, wherein the components are administered sequentially/in tandem (e.g., Barnes et al., 2013; Fiatarone et al., 2014; Law et al., 2014; Wang et al., 2014; Rahe et al., 2015; Karssemeijer et al., 2017; Lipardo et al., 2017), which can be contrasted with:
- “dual-task” interventions, wherein components are administered simultaneously, but typically are separate tasks (e.g., reciting numbers backwards while walking; Schaefer and Schumacher, 2011; Coelho et al., 2013b; Forte et al., 2013; Theill et al., 2013; Bherer, 2015; Eggenberger et al., 2015; Yokoyama et al., 2015; Desjardins-Crépeau et al., 2016), which can be further contrasted with:
- interactive interventions, as in exergaming, in which the actions in one realm affect the other (e.g., pedaling and steering controls progress in a virtual world and attainment of goals; Anderson-Hanley et al., 2012; Maillot et al., 2012; González-Palau et al., 2014; Schoene et al., 2014; Bamidis et al., 2015; Styliadis et al., 2015; Eggenberger et al., 2016; Lauenroth et al., 2016; Wang et al., 2016; Stanmore et al., 2017).

Indeed, there is a vast literature on “dual-task” experiments and interventions (Kramer et al., 1985; Hiyamizu et al., 2012; Kramer and Wong et al., 2015; Hosseini et al., 2017), which sometimes focus on the limitations of human abilities to manage two separate (non-interactive tasks) at once (e.g., walking and counting numbers backwards). Traditionally, the aim of dual-task training might focus on interference effects (Al-Yahya et al., 2011) and improving physical outcomes (e.g., decreasing falls) for impaired individuals (e.g., post-stroke), especially while managing dual tasks. More recently, studies have examined cognitive outcomes in dual tasks wherein there is more naturalistic interactivity (as in the cybercycle scenario above; Anderson-Hanley et al., 2012; as well as other interactive modalities such as dance: Foster, 2013; Kattenstroth et al., 2013; Dhami et al., 2015; Schoene et al., 2015; Burzynska et al., 2017; Marquez et al., 2017; Müller et al., 2017; Rehfeld et al., 2017). These recent studies seem to be converging on a similar cognitive-enhancing phenomenon that lends empirical support to the synergistic hypothesis outlined above and in part, elsewhere (Fissler et al., 2013). Recent reviews of the literature on the cognitive benefits of exergaming for older adults conclude the preliminary evidence is promising (Smith et al., 2010; Schoene et al., 2013; Bamidis et al., 2014; Barry et al., 2014; Chao et al., 2014; Ogawa et al., 2016; Zhu et al., 2016; Zilidou et al., 2016), but further research of the effects of these interactive interventions and the impact of specific components (e.g., passive stimulation vs. active cognitive training, aerobic vs. non-aerobic activity, etc.) is necessary.

We have previously hypothesized that the benefits of interactive physical and cognitive exercise may increase with increased mental effort and our lab published a pilot trial as a precursor to this present RCT, the Aerobic and Cognitive Exercise Study pilot (ACES-pilot; Barcelos et al., 2015). The ACES-pilot sought to investigate whether greater cognitive challenge while exergaming would yield differential outcomes in executive function and generalize to everyday functioning. Sixty-four community-dwelling older adults (mean age = 82) were randomly assigned to pedal a stationary bike, while interactively engaging on-screen with: (1) a low cognitive demand task (pedaling a steering along a virtual bike tour, referred to as *exer-tour*), or (2) a high cognitive demand task (pedaling and steering to chase dragons and score points in a 3D video game, referred to as *exer-score*). Executive function (indices from Trails, Stroop and Digit Span) was assessed before and after a single-bout and 3-month exercise intervention. A significant group × time interaction after 3 months of exergaming (Stroop; among 20 adherents). Those in the high cognitive demand group performed better than those in the low cognitive dose condition. Self-reported everyday function improved across both exercise conditions. These pilot data indicated that for older adults, cognitive benefit while exergaming increased concomitantly with higher doses of interactive mental challenge (Barcelos et al., 2015). The present study aims to replicate and extend that finding to MCI.

### Neurobiological and neuroimaging effects of physical and cognitive exercise

It is our hypothesis that interactivity can produce greater cognitive effects due to synergistic processes that compound or magnify the benefit in a non-linear way, perhaps by activation and utilization of neurobiological substrate of the mind-body interface that is evolutionarily adapted for success in naturalistic tasks (in this case, goal-directed motion through 3D space). While theory and research connecting cognitive processes to neurobiological pathways triggered by exercise in humans is expanding rapidly, there are indications of important links with various biomarkers (e.g., brain-derived neurotrophic factor [BDNF], insulin-like growth factor [IFG-1]; Cotman et al., 2007; Knaepen et al., 2010), including expression of microRNA (miRNA) in circulating exosomes (Pusic et al., 2016; Bertoldi et al., 2017), and brain structure and function (e.g., gray and white matter regions: Colcombe et al., 2006; hippocampus; Thomas et al., 2012; Erickson et al., 2015; Sexton et al., 2016). Animal models have more plainly linked certain differential, compounding or synergistic cognitive benefits with neurobiological phenomenon when both physical and mental exercise are provided (e.g., in laboratory mice exposed to enriched environments with physical and/or cognitive challenges; Greenough et al., 1999; Churchill et al., 2002; Olson et al., 2006; Galvan and Bredesen, 2007; Fabel et al., 2009; Voss et al., 2013; Jessberger and Gage, 2014). These studies and others, detail a variety of mechanisms and impacts linking mental and physical exercise to improved neuronal and brain health (e.g., via increased cerebral perfusion, neurogenesis, angiogensis, synaptogenesis; Greenough et al., 1999; Gage, 2002; Trachtenberg et al., 2002; Tsai et al., 2016; Kleemeyer et al., 2017), even detailing specific differential benefits (Black et al., 1990; Olson et al., 2006; Suo et al., 2016) such as cell proliferation with physical exercise, and cell survival with mental exercise (Van Praag et al., 2005; van Praag, 2008).

### The present research

Since only a tiny fraction of older adults exercise at levels recommended by the ACSM and American Heart Association (AHA; Chodzko-Zajko et al., 2009), and physician recommendation appears to do little to change sedentary behaviors (Grandes et al., 2009), the novel exergame utilized in this study holds the promise of increasing compliance by virtue of the distracting components of the virtual reality environment and the interactive and challenging features of the videogame. Besides being a potentially effective way of engaging older adults in exercise, the present RCT augments prior research by exploring the neuropsychological benefits of interactive cognitive and physical training combined, rather than focusing on either alone (Colcombe and Kramer, 2003; Anguera et al., 2013), or in tandem, but lacking interactivity (Fabre et al., 2002; Oswald et al., 2006; Kraft, 2012; Suzuki et al., 2012; Hotting and Roder, 2013; Shatil, 2013; González-Palau et al., 2014; Satoh et al., 2014; Shah et al., 2014; Suo et al., 2016). By examining the neuropsychological effects some of the components separately (e.g., physical exercise and mental exercise alone), this study attempts to tease apart the unique contributions of each component to any benefit to cognition, as well as the potentially differential effect of low- vs. high-cognitive demands. The present research specifically examines the benefits of these behavioral interventions for community-dwelling older adults with or at risk for MCI and thus, attempts to extend previous findings to this more vulnerable population.

Finally, this RCT examines potential underlying mechanisms of effects of combined physical and mental exercise on cognition through the collection of neurobiological and neuroimaging data. Prior research has begun to clarify the role a number of biomarkers (primarily from serum, CNS, or saliva) that change in accord with exercise interventions and appear to be linked improvements in brain health that and/or promote improvements in cognition. For example, exercise has been shown to yield: increases in Brain-Derived Neurotrophic Factor (BDNF; Cotman et al., 2007; Coelho et al., 2013a; Leckie et al., 2014; Vaughan et al., 2014; Dinoff et al., 2016; Maass et al., 2016) and variable responses of insulin-like growth factor 1 (IGF-1; Lorens-Martín et al., 2009). Research linking traditional physical exercise and cognition in older adults has identified BDNF and IGF-1 as biomarkers linking cognitive function and healthy aging (Voss et al., 2010; Bellar et al., 2011; Erickson et al., 2012; Hotting and Roder, 2013; Phillips et al., 2014; Szuhany et al., 2014; Geerlings et al., 2015; Lara et al., 2015; Maass et al., 2016). BDNF plays an important role in neuronal growth and survival, it modulates neurotransmitters, and is involved in the cascade to promote neuronal plasticity, while IGF-1 has also been implicated as a mechanism in exercise-induced neuronal plasticity (Voss et al., 2010; Brown et al., 2013; Phillips et al., 2015; Maass et al., 2016). Cotman et al. (2007) has presented a model in which exercise induces activity of BDNF and IGF-1 in multiple pathways that facilitate: (a) learning, (b) neurogenesis, and (c) angiogenesis. Furthermore, some research has found increases in vascular endothelial growth factor with exercise (VEGF; Vital et al., 2014). While growth factors are typically said to increase with exercise, there are number of reports of decreases in inflammatory markers: C-reactive protein and Interleukin-6 (CRP and IL6; Thielen et al., 2016; Monteiro-Junior et al., 2017). Additionally, recent progressive research has also been examining the potential for circulating exosomes to serve as early markers of neuropathology, such as AD, to act as delivery vehicles for potentially reparative miRNA expressions, and even to play a positive role the neurbiological effects of exercise (e.g., Zhang et al., 2014; Kumar and Reddy, 2016; Pusic et al., 2016; Batistela et al., 2017; Bertoldi et al., 2017). Some promising research has pointed to miRNA-9 and miRNA-193 as linked to benefit in neurobiological processes such that they play a therapeutic role in dementia (e.g., Russell et al., 2013; Párrizas et al., 2015; Li et al., 2016; Riancho et al., 2017).

In sum, exercise appears to be able to trigger or enhance the function of complex cascades of neurobiological processes that have sometimes been linked to cognitive benefits or other expressions of brain or CNS health. It is likely that a number of those processes act on neurophysiological substrate to foster improved brain health that leads to improved cognition; some of those mediating phenonmena may even be visible through structural imaging.

Structural changes in certain regions of the brain have been found to follow exercise and are often linked to concomitant changes in cognition; for example, increases in the hippocampus (Lorens-Martín et al., 2009; Erickson et al., 2011), as well as the anterior cingulate cortex, dorsal lateral prefrontal cortex, and more broadly the prefrontal cortex (ACC, DLPC, and PFC; Gordon et al., 2008; Erickson et al., 2011; Weinstein et al., 2012; Curlik and Shors, 2013; Hayes et al., 2013; Nishiguchi et al., 2015; ten Brinke et al., 2015; Jonasson et al., 2017; Li et al., 2017). While the body of research has been growing with respect to linking physical exercise and cognition via biomarkers, scant literature has yet explored how these indicators and mechanisms react in the case of combined or interactive mental and physical exercise interventions in humans, wherein there might be somewhat differential or compounding beneficial effects given a two-for-one intervention (for a promising exception, see Eggenberger et al., 2016). The present study aims to address a number of these gaps and replicate or extend other findings in the literature.

### Hypotheses

**Figure 2:**
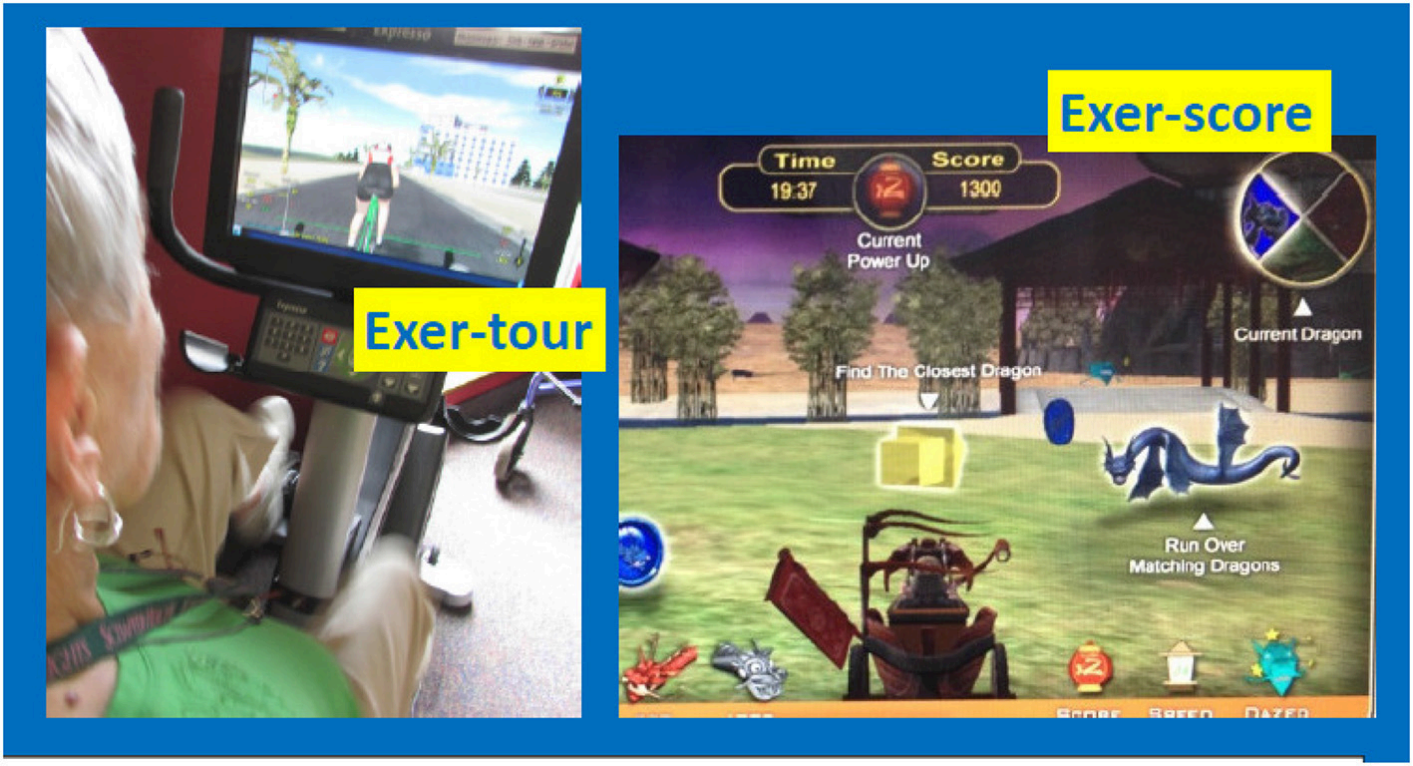
Exer-tour (relatively cognitively passive) vs. Exer-score (cognitively effortful). Exer-tour (pedaling controls speed on screen and progress along scenic bike paths; involves steering, but relatively passive compared to exer-score; for example, can't leave road or crash into anything or any rider which one can steer through; could cease steering without consequence other than tilted view, bike will follow curb). Exer-score (requires navigating in 360° radius to locate colored coins and matching colored dragons of varying speed/difficulty to steer through; the goal is to score points and strategy may be employed to avoid losing points by avoiding hazards, while also seeking out bonus points available via tagging specialized objects one can choose to explore). Former study participant demonstrating exer-tour condition; used with permission.

This study is a partial replication and extension of the ACES-pilot (Barcelos et al., 2015) and it was hypothesized that, similar to that which was found in that prior pilot study: physical exercise interactive with effortful cognitive challenge (exer-score; Figure 2), would produce greater cognitive benefit than physical exercise that was interactive with relatively passive cognitive processing (exer-tour). We expected this previously observed phenomenon to extend to MCI specifically. The primary aim of the trial was for community-dwelling older adults with or at risk for MCI to engage their assigned intervention regularly for 6 months (longer than the ACES-pilot, which extended only to 3 months). Baseline to 6-month intervention (exer-tour vs. exer-score) were hypothesized to yield these effects:

1. executive function would increase significantly after either exer-score and exer-tour (within subjects main effect)
2. executive function would increase significantly more for exer-score (high mental challenge) than for exer-tour (low mental challenge; between subjects interaction effect)
3. effects of either exer-score or exer-tour would exceed that of game-only
  - additionally, a comparison would be made with archival/normative exercise-only data (available only for a 3-month window, this trial's midpoint)
4. salivary biomarker changes expected to be correlated with improved cognition and exercise dose included:
  - increased growth factors: BDNF, IGF-1, and VEGF
  - decreased inflammatory markers: CRP and IL-6
  - increased exosomal expression of miRNA-193 and miRNA-9
5. neuroimaging changes expected to be associated with improved cognition and exercise dose included:
  - increased gray matter volume in the ACC, DLPFC, hippocampus, and PFC.

## Methods

### Participants

**Figure 3:**
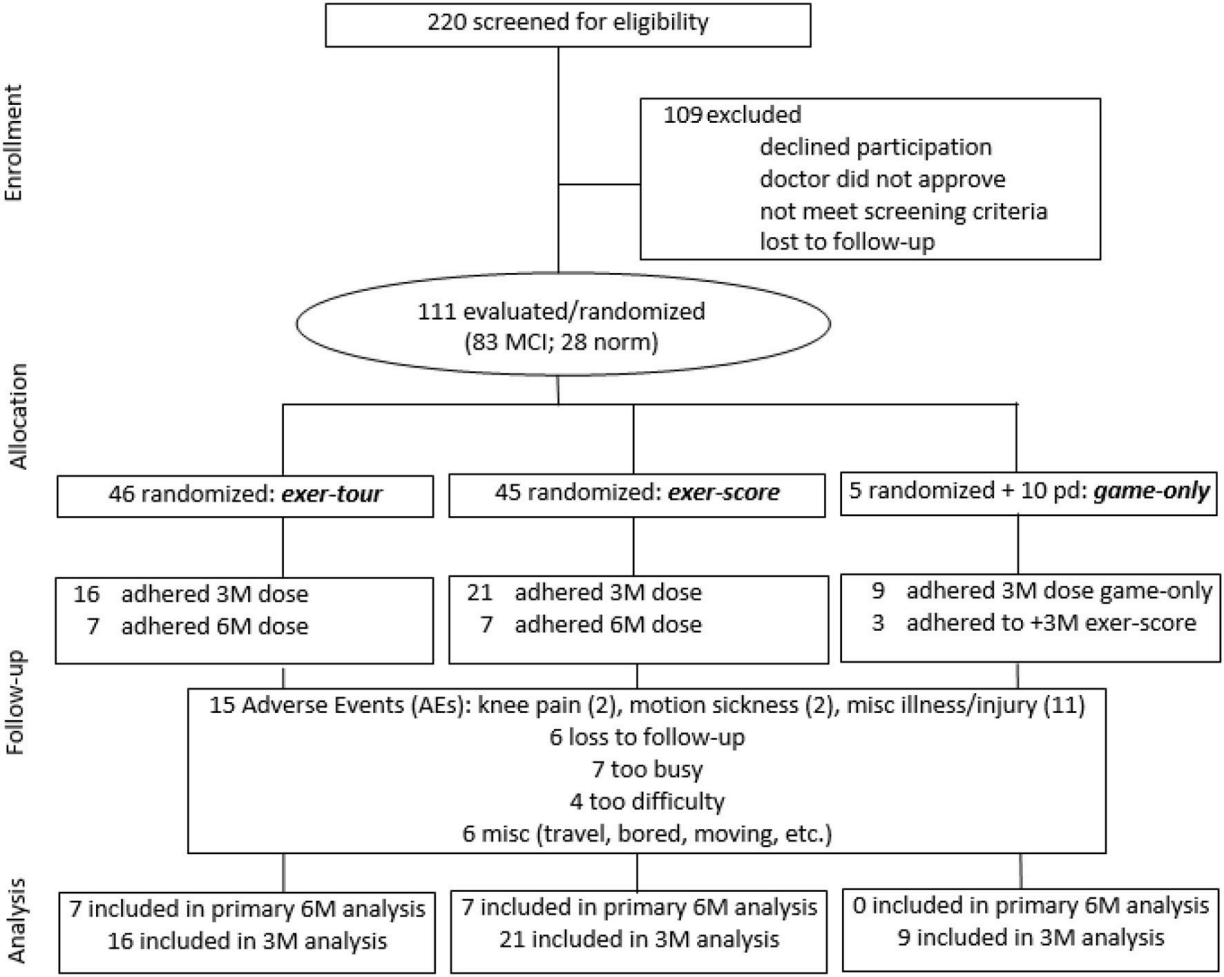
CONSORT flow diagram showing participant enrollment, randomization, and status in trial and analysis.

All study procedures were approved by the appropriate Institutional Review Boards (i.e., including the PI's institution and the three medical centers where the study was conducted; see section Author Notes). Older adults with a diagnosis of MCI were initially sought via referrals from neurology and neuropsychology at three medical centers in a specific region of upstate New York where participants were to exercise (using a cybercyle placed at each center's physical therapy clinic). However, given difficulties enrolling patients (especially due to travel requirements, but also due to challenges targeting patients with MCI before progression to dementia), further IRB approval was obtained to expand recruitment by locating additional cybercycles at several more locations around the region (e.g., retirement communities, YMCAs, etc.) for a total of 14 sites. Enrollment was thus expanded to include self-referred community-dwelling older adults, aiming to include especially those that might be undiagnosed (because symptoms had not affected function), but yet who might meet criteria for, or could be said to be at risk for MCI (e.g., thus those that would meet screening criteria for MCI, herein referred to as “screened as MCI” or sMCI^2^). Participants were recruited using fliers, newspaper ads, and information sessions. The timeline for the conduct of the study was 2014–2016. Volunteers (*n* = 220; see CONSORT Figure 3 for details) were screened by phone and were excluded if they had known neurologic disorders (e.g., Alzheimer's, Parkinson's, or seizures) or functional limitations that would restrict participation in cognitive testing or exercise. After reviewing the informed consent form (ICF) and answering all questions, a signed document was obtained from all participants and their surrogate, if appropriate, based on the Impaired Decision Making Capacity screen (IDMC; Karlawish, 2008). Physician approval (PCP and if applicable, cardiologist) was obtained for all participants to engage in exercise as assigned. Enrollees (*n* = 111) were community-dwelling older adults with a mean age of 78.1 (*SD* = 9.9), who were predominantly female (66%), well educated (average years of education = 16.2, *SD* = 2.4) Causasians (three individuals self-reported minority ethnic/racial status). Participants scored on average 23.7 (*SD* = 3.1) on the Montreal Cognitive Assessment (MoCA; 30-point multidimensional scale, with higher scores indicative of better global cognitive function; Nasreddine et al., 2005; Freitas et al., 2012; Julayanont et al., 2014), such that 75% of enrollees were categorized at meeting screening criteria for sMCI (“screened as MCI” based on MoCA < 26; Nasreddine et al., 2005).

To provide an additional point of comparison of the relative effect of physical exercise alone vs. interactively combined with mental exercise, archival data was culled from our prior Cybercycle Study. In that study, 33 participants were randomly assigned to a traditional exercise or “pedal-only” condition in which participants pedaled a stationary bike (in many cases the same model as used in the present study), but without the virtual reality display (see below for between group comparisons at baseline; for additional details see Anderson-Hanley et al., 2012, 2014; Dimitrova et al., 2016).

#### Baseline comparisons between groups

Between the three randomly assigned groups in the present study (exer-tour, exer-score, and game-only; *n* = 46, 45, 20, respectively), cognitive measures revealed that the groups were comparable (no significant differences) on: overall cognitive function (MoCA), rate of sMCI, and performance on individual tests of cognition including: executive function, visual spatial skill, and memory (verbal, nonverbal, list, story, immediate or delayed).

On behavioral measures, those who were in the game-only condition were somewhat less motivated to be in the study, were not as ready for exercise, and reported they were more sedentary (recall that some participants were randomly assigned to game-only, but others were recruited specifically to the condition, including with remuneration, due to a high dropout rate once randomly assigned participants learned they would not be engaging in a physical exercise component). However, self-reported cognitive engagement (both recent and lifetime), was similar across all three groups.

Baseline comparisons between the four groups, including the above three, but also a pedal-only condition (archival control group data from our prior study noted above; *n* = 33), revealed that all four groups were similar (no significant differences) on: the three primary outcome measures of executive function to be the focus of our study (i.e., ratio scores for Digits, Trails, and Stroop; see below for details). The four groups were also comparable in proportion of men and women and BMI, but the pedal-only condition was significantly older and had fewer years of education^3^ (confirming use of age and education covariates in statistical analyses).

### Procedures

Participants were initially randomly assigned to one of three conditions for 6 months: (1) exer-tour: physical exercise interactive with relatively passive, low cognitive load, virtual scenic bike tour; or (2) exer-score: physical exercise interactive with a relatively effortful, high cognitive demand, videogame (see Figure 2 for sample screen shots); or (3) game-only: the same videogame operated by a joystick or keyboard (no physical exercise was required). Exercise participants pedaled a virtual reality enhanced, recumbent stationary bike (Expresso S3R, from Interactive Fitness Holdings, LLC). Individuals in the exer-tour condition pedaled through various virtual scenic bike tours while steering along the path (but without significant consequence for not steering; that is the bike would coast along the curb and one could pass through other riders fluidly). Those in the exer-score condition pedaled through a scenic landscape where the goal was to score points by collecting different colored coins and corresponding colored dragons. Throughout the videogame (either exer-score or game-only), participants could navigate through bonus items to increase their speed and score, while avoiding penalties (e.g., steering into water). The exer-tour condition was presumed to require less mental engagement than the videogame conditions, as the game called for scoring and thus, potentially effortful planning, tracking, multi-tasking, and strategizing (a validity check of this assumption was confirmed by those in the exer-score condition who reported expending significantly greater “mental effort” than those in the exer-score condition; see Table 1). Participants were provided “thank you gifts” (e.g., water- bottle, mug, etc.) at each of evaluation (baseline, 3-month, and 6-month).

**Table 1:** Exer-tour vs. exer-score baseline demographics for adherent completers (0–6M).

	**MCI adherents to prescribed dose of 6-month intervention**													**MCI**
	**Exer-tour**			**Exer-score**			**ANOVA**	**Combined adherents**			**Non-adherents**			**ANOVA**
	**Ave**	***SD***	***n***	**Ave**	***SD***	***n***	***p*^a^**	**Ave**	***SD***	***n***	**Ave**	***SD***	***n***	***p*^a^**
**DEMOGRAPHICS**														
Age	80.9	12.3	7	75.4	9.83	7	0.38	78.1	11.0	14	80.9	8.2	54	0.31
Education (yrs)	14.9	2.3	7	16.6	2.76	7	0.23	15.7	2.6	14	16.1	2.5	52	0.61
Sex (% female)	57%			43%			0.63	50%			69%			0.20
Est IQ (NAART)	128.5	11.9	7	136.8	5.26	7	0.12	132.7	9.8	14	134.3	7.3	54	0.49
Cog fn (MoCA)	21.6	2.7	7	22.0	3.21	7	0.79	21.8	2.9	14	22.6	2.0	54	0.20
Retired	100%		7	100%		7	ns	100%			86%			0.15
BMI	24.8	3.9	7	26.3	2.73	7	0.43	25.6	3.3	14	26.9	3.7	54	0.24
Cog activities (CAS)	17.9	4.5	7	20.1	3.98	7	0.34	19.0	4.3	14	19.3	8.3	54	0.89
Experience bike	2.4	0.79	7	2.3	0.76	7	0.74	2.4	0.74	14	2.1	0.84	52	0.39
Experience computers	2.1	1.1	7	3.1	1.07	7	0.11	2.6	1.2	14	2.6	1.2	52	0.98
Experience videogames	0.43	0.79	7	1.3	0.76	7	0.06	0.9	0.86	14	0.69	0.85	52	0.52
Motivated	2.6	0.53	7	2.4	0.53	7	0.63	2.5	0.52	14	2.7	0.47	51	0.20
PAR	0.86	0.69	7	0.86	0.69	7	1.00	0.9	0.66	14	1.1	1.0	50	0.31
Physical activity level (SRPA)	2.9	1.3	7	3.6	0.98	7	0.28	3.2	1.2	14	2.9	1.2	47	0.43
Functional disability (FAQ)	8.1	9.0	7	1.6	1.81	7	0.08	4.9	7.1	14	2.8	4.7	49	0.06
**VALIDITY CHECKS**														
Average num rides/wk	3.9	1.2	7	4.0	1.1	7	0.86							
HR average	102.9	13.6	7	108.2	22.1	7	0.60							
Mental effort (self-report)	1.8	0.75	6	3.1	0.69	7	**0.007**							

a *Comparison between groups*.

*b Comparison with baseline*.

*c Repeated meas ANCOVA controlling for age and education*.

*Bold value indicate statistically significant (p ≤ 0.05)*.

Given difficulty retaining participants in the game-only condition (due to preference for active/physical exercise, boredom once started, etc.), the protocol was again adjusted, with IRB approval, to allow randomly assigned game-only participants to switch to the exer-score condition at the 3-month mid-point evaluation. Additionally, game-only participants were also recruited separately with modest periodic remuneration provided. Upon enrollment, participants were phone screened for eligibility and administered the Impaired Decision-Making Capacity structured interview (IDMC; Veterans Administration Medical Center (VAMC), 2007) and provided informed consent (co-signed by a surrogate or legally-authorized representative as applicable and/or per the IDMC). Once scheduled for an initial evaluation, participants were mailed the ICF, demographic, health and fitness history questionnaires. If they were willing, an MRI (3T) of the brain was scheduled. At the baseline evaluation, a saliva sample was collected (passive drool), and they were administered a neuropsychological test battery (specified below). Individuals were trained in the use of the equipment for their assigned condition and underwent a single bout of exercise for 20 min. Participants were asked to maintain a target heart rate level, which was calculated using the Karvonen equation (McAuley et al., 2011) and measured via steering hand-grips built into the cybercycle (displayed on screen for participant monitoring and captured by onboard computer for later data extraction and analysis). Post-testing was conducted following the single bout^4^ (SB) of exercise.

Following the baseline (BL) evaluation, individuals were instructed to exercise for at least 20 min at least twice a week, and to gradually increase exercise duration to 45 min and frequency to at least three to five times per week until the 3-month (3M) mid-point evaluation, and then maintain that pattern though a 6-month (6M) final evaluation. Participants were instructed to aim toward their individualized specific target heart rate (per above) throughout the intervention period. Participants were also asked to document their exercise sessions on log pages provided in a binder and used to calculate adherence statistics (e.g., frequency, heart rate average, etc.); additionally, the computer onboard the cybercycle captured similar data which was used for spot verification. A final evaluation, including repeat neuropsychological, biomarker, and neuroimaging assessments, was conducted at the end of the 6-month intervention period.

## Measures

### Primary measures

#### Cognitive function

Paper and pencil tests comprised the battery and alternate forms of cognitive tests were utilized at each evaluation to minimize practice/learning effects from serial testing. Cognitive testing was done at baseline (BL) before starting the intervention, after the 20-min training single-bout session, at a 3-month (3M) mid-point, and after concluding the 6-month (6M) intervention period.

#### Executive function (primary outcome)

Three tests were selected to assess aspects of executive function, which has been shown to be a complex, yet overarching domain of cognitive function. The following three tests were chosen as they are thought to tap overlapping, yet distinct components of executive function, particularly mental flexibility (i.e., Stroop for inhibition, Trails for set shifting, and Digit Span for working memory; Wecker et al., 2000; Strauss et al., 2006). Executive function is critical cognitive function for maintaining independence in later life and thus an important focus of this study's intervention efforts (Pereira et al., 2008; Marshall et al., 2011).

##### Stroop (Van der Elst et al., 2006)

A short, 40-item version of Stroop was administered. Colored squares (red, green, blue) were presented in rows first (Stroop A), followed by those color words typed in black ink (Stroop B), followed by incongruent color words (Stroop C; in which participants were asked to name the color of the ink while ignoring the written word). A ratio was computed to isolate the executive function component of the task (Stroop A/C; Lansbergen et al., 2007). Among older adults, the ratio score has reasonable test-retest reliability (0.68) over 1–2 months (Ettenhofer et al., 2006) and strong test-retest reliability (0.80) over 2 weeks using the shortened version used herein (Houx et al., 2002). Higher ratios indicate better executive function.

##### Color trails (D'Elia et al., 1996)

Color Trails 1, requires participants to connect numbered circles in ascending order. Color Trails 2, requires individuals to connect numbered circles in consecutive order while also alternating the color of the circle (pink or yellow). Following the pattern set by Lansbergen et al. (2007) above of isolating the executive function component by dividing basic and faster processing speed by the slower interference trial, a ratio score (Trails1/2) was computed. Reliability and validity are adequate (D'Elia et al., 1996). Higher ratios represent better executive function.

##### Digit span (Strauss et al., 2006)

Digit Span Forward requires participants to first listen to a list of numbers and repeat them, with the string length increasing to the maximum of their ability. Digit Span Backward, requires repeating a string of numbers in reverse order. Continuing the pattern above to isolate the executive function component, the ratio of the typically smaller sum of correct interference trials on Digit Span Backward, divided by the typically greater sum of correct basic attention trials on Digit Span Forward (DigitsB/F). Good reliability and validity have been reported for Digit Span (Strauss et al., 2006). An increase in the ratio was the desired outcome.

### Secondary measures (characterization of sample and possible additional outcomes)

#### Montreal cognitive assessment (MoCA; Nasreddine et al., 2005)

The MoCA was administered at baseline to characterize the sample as either normative aging or “screened as MCI” (sMCI; see details above). The MoCA consists of eight different subtests to assess overall cognitive impairment. Scores below 26 out of 30 were used to categorize sMCI (Nasreddine et al., 2005).

#### Ecological validity (EV; self-reported cognitive function; Klusmann et al., 2010)

In an attempt to measure any generalized, clinically relevant, effects of the interventions (beyond any training to the tests that might occur when using standardized, serial testing), a participant self-report of perceived cognitive function was utilized. Participants were asked about their self-perceptions and beliefs related to their everyday functioning in regards to their cognitive functioning (e.g., memory and concentration). The Ecological Validity Questionnaire (Klusmann et al., 2010) was administered at BL, 3M and 6M. This measure consists of eleven statements that ask about participants perceptions of memory, concentration, and everyday function. Using a 5-point Likert scale (1 = absolutely wrong / bad to 5 = absolutely true / good), participants rated how much each of the statements described their behavior over the past 2 weeks. The total score can range from 11 to 55, with higher scores representing perceptions of better cognitive functioning.

#### Verbal memory (alzheimer's disease assessment scale; ADAS word list—immediate and delayed recall; Harrison et al., 2007; Podhorna et al., 2016).

Participants are shown a list of 11 words on cards and they recall as many as they are able immediately and also after a delay interval. The number of errors/omissions comprises the score, so lower scores are better.

#### Get-up-and-go test (Podsiadlo and Richardson, 1991)

Participants rise from sitting, walk 10 feet, turn around and return to sitting position. The time it takes to complete the task is the score. Lower scores are better.

#### Other measures include

The Physical Activity Readiness Questionnaire (PAR-Q; 0–7, higher scores indicates barriers to readiness; NASM) and Self-reported Physical Activity (SRPA); mental and physical effort thermometers; Exercise Induced Feeling Inventory (EIFI); the Confusion and Vigor subscales of the Brunel Mood Scale (Terry et al., 2003); experience of Flow (Payne et al., 2011), and other neuropsychological measures (e.g., Rey-O, NAART, etc.) not expected to be affected by the intervention, but included for separate use in full characterization of enrollees for later cross-sectional analyses. Due to the focus of this report on a priori hypotheses, and that the small adherent sample available for final analysis and circumscribes statistical power, some of these measures will be reported separately in cross-sectional or other limited analysis.

### Exploratory measures

The following biomarker and neuroimaging data were collected from some willing and able participants, who comprised a small subset of the enrolled participants. Due to the small and incomplete sample, we report these as pilot data to examine comparisons that were planned a priori and provide tentative findings, which may be useful in directing future research.

#### Biomarkers

Saliva samples (passive drool per Salimetrics protocol) were collected at: BL, SB, 3M, and 6M from willing and able participants. Samples were centrifuged, with a portion reserved for protein analyses and a portion further processed for exosome analyses. Exosomes were isolated from whole saliva via differential ultracentrifugation as described in previously published protocols (Thery et al., 2006; Gallo and Alevizos, 2013; Witwer et al., 2013). Briefly, whole saliva was subjected to differential centrifugation steps at 300 × g, 1,500 × g, 17,000 × g, and 160,000 × g. The exosome pellet was suspended in 1X PBS. All samples were stored at −80°C until analyses were conducted.

#### Protein assays

Frozen samples were shipped overnight on dry ice for analysis (to RayBiotech, Inc., Norcross, GA). Protein concentrations of BDNF, CRP, IGF-1, IL-6, and VGEF were obtained via ELISAs (e.g., Mandel et al., 2011). Each patient sample was run in duplicate using 100 ml of plasma diluted by a factor of 2. A commercial multiplexed sandwich ELSIA-based array was used (Quantibody custom array, RayBiotech Inc., Norcross, GA, USA). All of the samples were tested using a panel of cytokines per above. The antibody array is a glass-chip-based multiplexed sandwich ELISA system designed to determine the concentrations all cytokines simultaneously. One standard glass slide was spotted with 16 wells of identical biomarker antibody arrays. Each antibody, together with the positive and negative control, was arrayed in quadruplicate. The samples and standards were added to the wells of the chip array and incubated for 3 h at 4 uC. This was followed by three to four washing steps and the addition of primary antibody and HRP-conjugated streptavidin to the wells. The signals (Cy3 wavelengths: 555 nm excitation, 655 nm emission) were scanned and extracted with an Innopsys laser scanner (Innopsys, Carbonne France), and quantified using Quantibody Analyzer software (Ray Biotech Inc). Each signal was identified by its spot location. The scanner software calculated background signals automatically. Concentration levels, expressed in picograms per milliliter (pg/ml), were calculated against a standard curve set for each biomarker from the positive and negative controls.

#### Exosome analyses

Frozen samples were shipped overnight on dry ice for analysis (to VY at Oklahoma State University). The exosome/PBS solution was lyophilized for 9 h and RNA extraction was performed using Trizol™ reagent (Life Technologies®, catalog # 15596026.PPS) following the manufacturer's protocol. A fixed volume of 1,000 ng RNA was reverse-transcribed using the High Capacity cDNA Reverse Transcription Kit (Appled Biosystems, catalog #: 4368814). PCR forward and reverse primers for the human miRNA-193 (forward: CTTTTGGAGGCTGTGGTCTCAGAATC; reverse: CCAGTTGGATAAAACATAAACTCATCTCGCC) and miRNA-9 (forward: AGGCGGGGTTGGTTGTTATCTTTG; reverse: CTAGCTTTATGAAGACTCCACACCACTCATAC) were used for real-time quantitative RT-PCR (qRT-PCR) amplification. miRNA-193 and miRNA-9 expression was normalized against the U6 housekeeping miRNA (forward: GTGCCTGCTTCGGCAGC; reverse: TATGGAACGCTTCACGAATTTGCGTG).

The real-time qRT-PCR was performed using SYBR® Select Master Mix (ThermoFisher, cat #: 4472919). 100 ng of cDNA was loaded per well and amplified for 45 cycles using Opticon® 2 real-time PCR instrument (Biorad, model #: CFB-3220) operated by Opticon Monitor 3 software. All samples and references were run in triplicate and each well contained 20 μL total volume. Raw florescence readings were directly imported into, and baseline corrected with, LinregPCR software package (Version 2016.1). Linear regression was performed on the baseline-corrected data to calculate efficiency using a common window-of-linearity for each primer pair (Ruijter et al., 2009). Between-session variations were estimated using a maximum likelihood approach (Ruijter et al., 2006) and inter-plate variability was minimized using factor correction calculated using Factor qPCR software (Version 2016.0). Taking into account the reaction efficiency of each primer set, we used REST 2009 software package (Version 2.0.13, QIAGEN, Valencia, CA, USA) to calculate the relative RNA expression ratios in samples by a Pair Wise Fixed Reallocation Randomization model with 8000 iterations and a combination of randomization and bootstrapping techniques (Pfaffl et al., 2002).

#### Neuroimaging

Brain imaging T1-weighted 3D MP-RAGE axial images were acquired with a Siemens MAGNETOM Verio 3T MRI scanner running syngo MR B19 software, using an 32-channel bodycoil using the following parameters: 0.5 × 0.5 × 1.3 mm^3^ voxel size; 256 × 256 matrix; 25 cm field of view; flip angle = 8°; echo time = 2.9 ms; repetition time = 1,640 ms; inversion time = 828 ms. Gray matter (GM) volume was analyzed using FreeSurfer software v. 5.3.0^5^ (Fischl et al., 2002), in a Linux environment, as described in detail previously (Destrieux et al., 2010). Briefly, cortical reconstruction and volumetric segmentation require the initial motion correction and averaging of T1 images, signal intensity normalization, the removal of non-brain tissue, automated Talairach transformation and segmentation of gray and white matter^6^, and the parcellation of the cortex into well-described units, using gyral and sulcal landmarks for automatic differentiation. Each segmented brain volume was then inspected visually for processing errors which can occur during automation, and these were corrected using manual edits and re-processed. The Longitudinal Stream pipeline, available in Freesurfer v.5.3.0 (see website^7^), was used to create an unbiased within-subject template for the two timepoints in the present study (Reuter et al., 2012). Moreover, we expect good reliability and a minimal risk of bias-introduction with the automated pipeline, given that the within-subject scans took place in the same scanner using the same parameters and our focus was on cortical ROIs, which show somewhat better scan-rescan reliability than subcortical structures (e.g., Mills and Tamnes, 2014).

Given the literature cited above and a prioi hypotheses, the regions-of-interest (ROIs) included: hippocampus, dorsolateral prefrontal cortex (DLPFC), prefrontal cortex (PFC) and anterior cingulate cortex (ACC)^8^. Freesurfer does not directly extract these ROIs as they are not singular regions of the brain (see discussion above as to why they are structurally and functionally significant in the present context). As such, we defined our ROIs by combining the following Freesurfer labels: DLPFC = frontal middle gyrus and sulcus from the Destrieux et al. (2010) atlas; ACC = rostral and caudal anterior cingulate cortices from the Desikan et al. (2006) atlas; PFC = ACC + medial orbitofrontal and transverse frontopolar regions (Domenech and Koechlin, 2015). The DLPFC ROI that was chosen is one of the most conservative and non-controversial groupings, although some others have included more medial and/or caudal regions in their work (Vijayakumar et al., 2014; Jonasson et al., 2017). The volumes extracted are a measure of total gray matter only (white matter was parcellated separately and excluded). For the final analysis, GM volumes of each region were extracted and considered as a ratio to total intracranial volume to account for variations in brain size. Given the small sample size, volumetric ratios were used in parametric partial correlations with cognitive outcomes (controlling for age and sex).

#### Statistical analyses

Data were analyzed using SPSS version 23 for Windows (IBM Corporation). The primary goal of the RCT was to examine, for those with sMCI, the possible differential effects (via neuropsychological, neurobiological, and neuroimaging measures) of long-term use of different combinations of physical and cognitive exercise (exer-tour vs. exer-score). Analyses focused on those sMCI participants that adhered to the protocol (approximating the minimum prescribed dose by averaging 3 × /wk for 6 months, allowing for 2 weeks of vacation, illness, or equipment failure, in each of the 3-month evaluation windows). Given the small sample of completers, there was concern about insufficient power since a priori power analysis suggested a larger sample was indicated (Faul et al., 2007). Additionally, small samples often violate assumptions of standard parametric tests. However, the data for the primary outcome variables was found to be normally distributed (Shapiro-Wilk tests for StroopA/C, Trails1/2, and DigF/B were 0.52, 0.71, 0.16, respectively; Normal Q-Q plots were also reviewed which appeared to be normally distributed, with data following a linear pattern) and error variances were consistent with parametric assumptions (Levine's tests were not significant). Furthermore, given the importance of covariates (more readily implemented in parametric tests), along with the possibility that effects could be magnified in the sMCI sample, and following guidance in the literature on statistical strength of ANCOVA above nonparametric approach, especially in clinical trials (wherein a focus on change from baseline facilitates a trend toward normality; Vickers, 2005), it was decided to proceed with standard parametric analyses. Repeated measure ANOVAs, controlling for age and education were used to examine group (exer-tour vs. exer-score) × time (BL vs. 3M and 6M) interaction effects on the primary outcome measures of executive function. Covariates entered into the repeated measures analyses included: age and education, due to links to outcomes noted in the theoretical and empirical literature (Hannay and Lezak, 2004; Lam et al., 2013). Paired *t*-tests were used to evaluate within group change from baseline to the 3-month mid-point, and baseline to conclusion of the 6-month intervention.

Partial correlations, controlling for age and sex (based on prior literature demonstrating links to secondary outcomes; e.g., BNDF decreases with age: Lommatzsch et al., 2005; brain region variability with sex: Ruigrok et al., 2014), were conducted to explore the relationship between cognitive change and possible corresponding change in biomarkers (proteins and exosomes) and neuroimaging regions of interest, per above hypotheses. Further correlations were conducted to explore relationships between baseline memory and exercise dose as represented by ride frequency.

## Results

### Effect on primary outcome: executive function

#### Full (6-month) intervention (exer-tour vs. exer-score)

**Figure 4:**
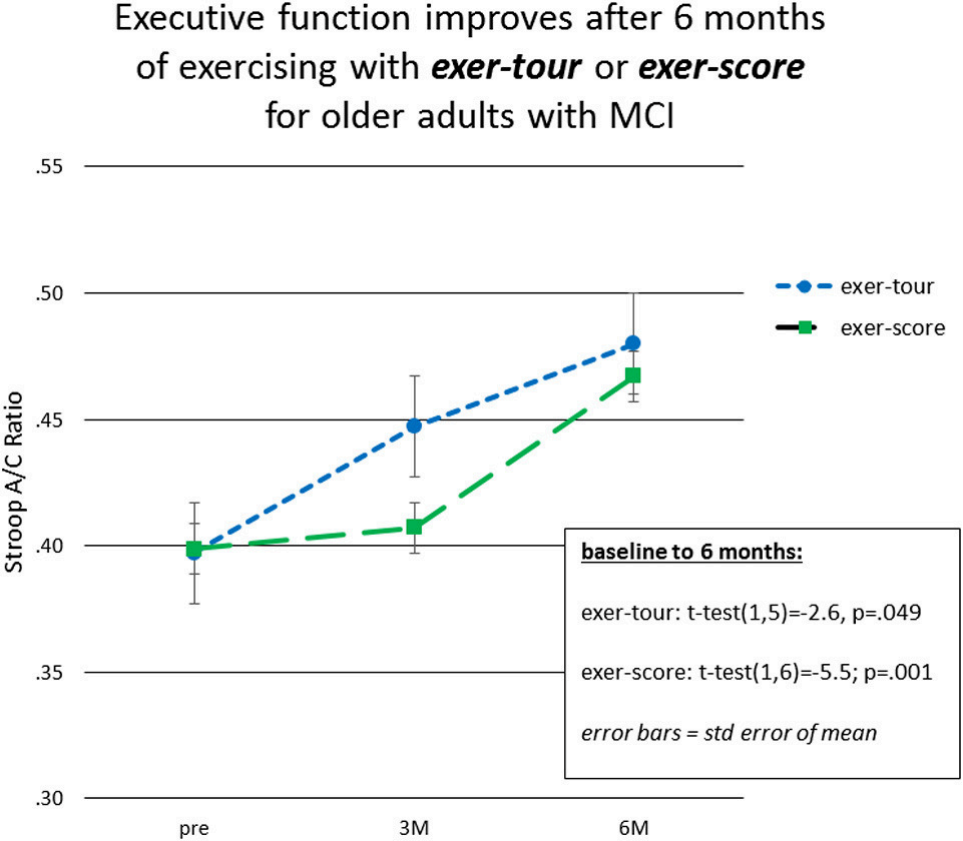
Exer-tour vs. exer-score for sMCI adherents 0–6M.

Fourteen participants who met criteria for sMCI (MoCA < 26) were adherent to the protocol approximating the minimum assigned weekly average of 3 × /week over 6 months. Half had been randomized to exer-tour and half to exer-score. After 6 months of interactive physical and cognitive exercise, the repeated measures ANCOVA omnibus test of executive function measures revealed a non-significant group x time interaction effect, *F*_(3, 7)_ = 0.85, *p* = 0.51, η_*p*_^2^ = 0.27. Univariate tests for StroopA/C, Trails1/2, and DigB/F were also not significant (*p* = 0.82, 0.11, 0.99, respectively). Paired *t*-tests were conducted to examine the change within groups over 6 months and StroopA/C improved significantly in both the exer-tour, *t*_(1, 5)_ = −2.6, *p* = 0.049, and exer-score conditions; *t*_(1, 6)_ = −5.5; *p* = 0.001 (Figure 4); Trails1/2 and DigB/F did not change significantly in either group (Table 2). Paired *t*-tests of the change from baseline to the mid-point (3M) evaluation revealed no significant differences between the exer-tour and exer-score conditions at that point among sMCI participants (*n* = 14) that were adherent through 6 months (Table 2). sMCI participants who had been assigned to either exer-tour or exer-score and were not adherent over 6 months (*n* = 54) did not differ significantly from adherent sMCI participants (*n* = 14) on any baseline variables (including demographics, cognitive, physiological and functional variables; Table 1).

**Table 2:** Exer-tour vs. exer-score for primary neuropsychological outcomes (0–3M−6M).

	**MCI adherents to prescribed dose of 6-month intervention**												**MCI**			**ANOVA**
	**Exer-tour**				**Exer-score**			**ANOVA**	**Combined adherents**				**Non-adherents**			
	**Ave**	***SD***	***n***		**Ave**	***SD***	***n***	***p*^a^**	**Ave**	***SD***	***n***		**Ave**	***SD***	***n***	***p^*a*^***
**PRIMARY OUTCOME**																
**Baseline executive function**^a^																
Color Trails $\frac{1}{2}$	0.49	0.17	7		0.47	0.10	7	0.85	0.48	0.13	14		0.48	0.21	54	
Stroop A/C	0.40	0.12	7		0.40	0.14	7	0.98	0.40	0.12	14		0.44	0.12	54	
Digits B/F	0.53	0.13	7		0.59	0.21	7	0.56	0.56	0.17	14		0.61	0.16	54	
				***t*****-test**				***t*****-test**				***t*****-test**				**(grp × time)^c^**
**3-Month executive function**				***p**^*b*^*				***p**^*b*^*				***p**^*b*^*				***P***
Color Trails $\frac{1}{2}$	0.44	0.11	7	0.41	0.44	0.07	7	0.34	0.44	0.09	14	0.20				0.89
Stroop A/C	0.45	0.06	7	0.25	0.41	0.15	7	0.68	0.43	0.11	14	0.21				0.46
Digits B/F	0.53	0.23	7	0.97	0.61	0.12	7	0.65	0.57	0.18	14	0.77				0.93
**6-Month executive function**																
Color Trails 1/2	0.38	0.12	6	0.10	0.48	0.17	7	0.83	0.44	0.15	13	0.32				0.11
Stroop A/C	0.48	0.11	6	**0.049**	0.47	0.15	7	**0.001**	0.47	0.13	13	**0.0002**				0.82
Digits B/F	0.60	0.15	6	.38	0.64	0.16	7	0.50	0.62	0.15	13	0.26				0.99

a *Comparison between groups*.

b *Comparison with baseline*.

c *Repeated meas ANCOVA controlling for age and education*.

*Bold values indicates statistically significant (p ≤ 0.05)*.

#### Mid-point (3-month) intervention (exer-tour vs. exer-score vs. game-only vs. pedal-only)

**Figure 5:**
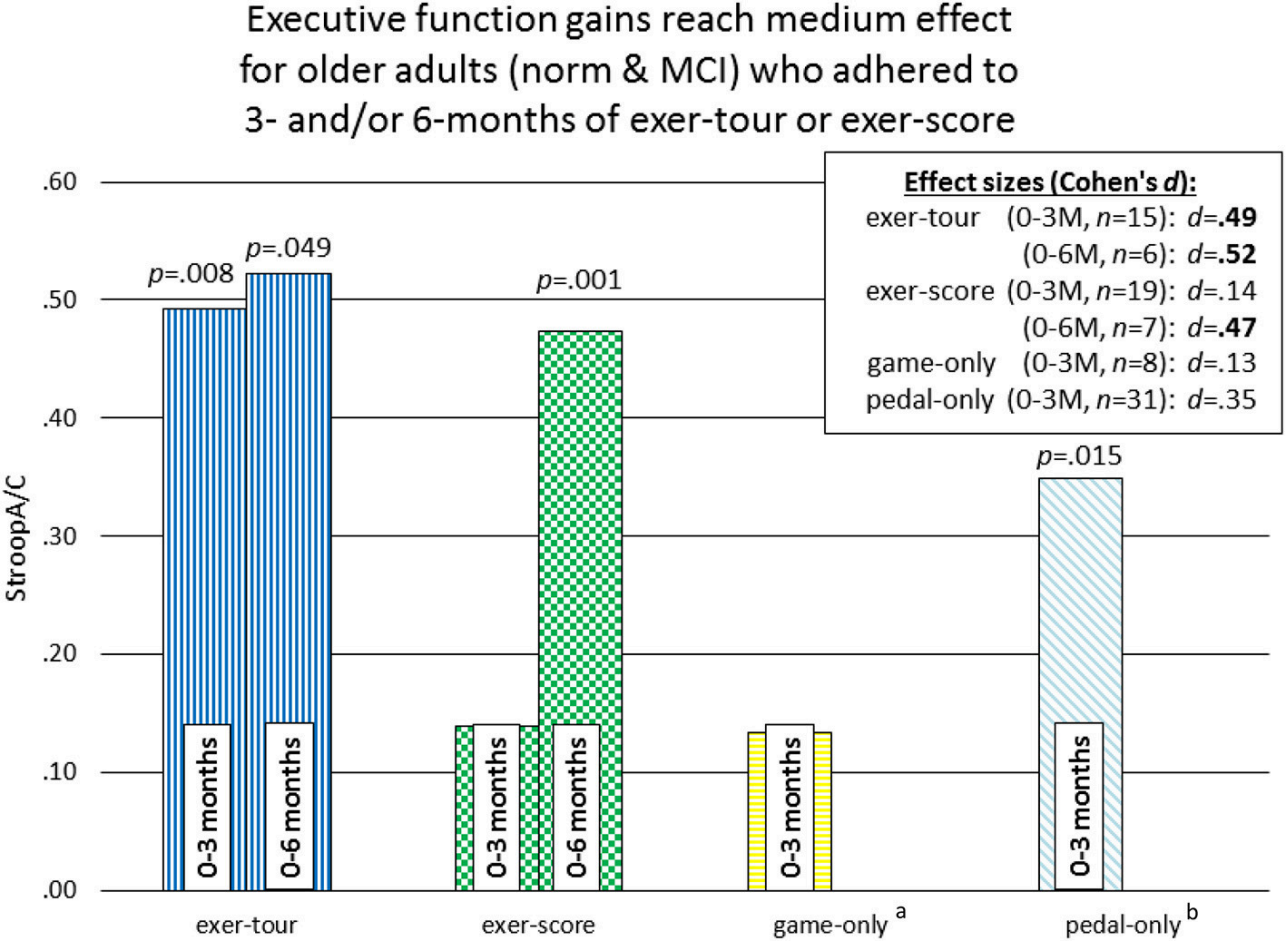
Comparison of effect sizes of components of interactive physical and cognitive exercise across four intervention conditions for 0–3M adherents. *p*-values represent significant change over time within-group (paired) *t*-tests. ^a^the game-only condition was initially randomly assigned, but subsequently recruited separately due to challenges with enrollment and retention (see section Methods for further details). ^b^the pedal-only comparison data is archival/perviously reported from our lab and was obtained during the Cybercycle Study (Anderson-Hanley et al., 2012), which examined only a 3-month interval and was collected from older adults in the same region, from many of the same retirement communities, and with the same measures of executive function; this data is included here as a point of comparaitve reference, illustrating the magnitude of effect from physical exercise alone.

Given the high level of attrition from the game-only condition, as noted above, a mid-course adjustment was made to enroll game-only participants into a 3M-only intervention window. Doing so provided some comparative data regarding the possible relative effect of the mental exercise component separate from physical exercise (videogame practice only). Additionally, we were able to extract comparative 3M control data from our prior Cybercycle Study regarding the effect of a pedal-only condition for older adults. These 3M analyses utilized all older adults (sMCI + normative^9^) that were adherent through 3M in their assigned condition (thus the n's for exer-tour, exer-score, game-only, and pedal-only were: 15, 19, 8, 31, respectively). Paired *t*-tests were conducted and revealed significant gains on StroopA/C by 3M for exer-tour and pedal-only, with statistically significant moderate effects (*d* = 0.49 and 0.35, respectively; Figure 5), while exer-score and game-only yielded non-significant small effects (*d* = 0.14 and 0.13, respectively). TrailsA/B and DigF/B did not change significantly. The 6M effect sizes for the two conditions that progressed to 6M (exer-score and exer-tour) are also included in Figure 4 and reveal that both the exer-tour and exer-score conditions yielded statistically significant moderate effects by 6M (*d* = 0.52 and 0.47, respectively); thus, the exer-score group seems to “catch up” to the exer-tour from 3 to 6M.

### Effect on secondary outcomes

#### Full (6-month) intervention (exer-tour vs. exer-score)

There were significant group x time interactions among the 14 sMCI adherents for: (1) immediate verbal memory (exer-tour improved significantly more than exer-score; *p* = 0.018), (2) self-report of everyday cognitive function (as assessed on the Ecological Validity Questionnaire), with exer-tour reporting significantly more improvement while exer-score reported decline; *p* = 0.046), and (3) physical ability (exer-tour increased significantly more than exer-score; *p* = 0.001; see Table 3).

**Table 3:** Exer-tour vs. exer-score for secondary outcomes: memory, everyday function (self-reported cognition and physical ability (0–6M).

	**MCI adherents to prescribed dose of 6-month intervention**												**MCI**			**ANOVA**
	**Exer-tour**				**Exer-score**			**ANOVA**	**Combined adherents**				**Non-adherents**			
	**Ave**	***SD***	***n***		**Ave**	***SD***	***n***	***p*^a^**	**Ave**	***SD***	***n***		**Ave**	***SD***	***n***	***p*^a^**
**SECONDARY OUTCOMES**								***t*****-test**								
**Baseline**								***p**^*a*^*								
Verbal Mem Immed (errors)	15.71	4.39	7		14.43	5.50	7	0.64	15.1	4.8	14		11.96	3.62	54	
Verbal Mem Delay (errors)	6.0	2.8	7		6.6	2.8	7	0.71	6.3	2.7	14		5.2	2.3	54	
Ecological Validity	35.5	7.9	7		40.2	9.8	7	0.34	37.8	8.9	14		41.6	7.9	50	
Physical ability (GUG)	12.7	2.2	7		10.3	1.7	7	**0.039**	11.5	2.3	14		12.7	3.5	54	
				***t*****-test**				***t*****-test**								**(grp × time)^c^**
**6-month**				***p***^b^				***p***^b^								***P***
Verbal Mem Immed (errors)	11.17	4.49	6	**0.003**	12.71	4.31	7	0.10	12.0	4.3	13	**0.001**				**0.018**
Verbal Mem Delay (errors)	5.5	3.0	6	0.53	5.1	2.9	7	**0.047**	5.3	2.8	13	**0.040**				0.15
Ecological Validity	41.3	7.3	6	0.20	36.6	7.2	7	0.12	38.8	7.3	13	0.84				**0.046**
Physical ability (GUG)	14.0	1.5	6	**0.009**	11.0	2.0	6	0.46	12.5	2.3	12	**0.013**				**0.001**

a *Comparison between groups*.

b *Comparison with baseline*.

c *Repeated meas ANCOVA controlling for age and education*.

*Bold values indicates statistically significant (p ≤ 0.05)*.

### Biomarkers^10^

Biomarker samples were sought from all participants, but not all were willing or able to provide saliva samples (data from completers was available from a maximum of *n* = 16 for analysis).^11^ Target analytes included: BDNF, CRP, IGF-1, IL-6, and VEGF. Due to low concentrations of IGF-1 in the samples collected, the assay results were below the limit of detection on too many samples and this assay was excluded from analyses. Partial correlations (controlling for age and sex; Table 4) were conducted to examine the change from baseline to 6M in biomarkers related to change in the primary outcome measure, executive function (StroopA/C) which, per above, changed significantly with the intervention. Similarly, change in biomarkers were correlated with a secondary outcome that was impacted significantly by both interventions, was verbal memory, and we chose to focus on delayed verbal memory specifically (ADAS errors), since one of the more salient cognitive functions to impact for MCI patients. Finally, the exercise dose, based on average number of rides, was examined for association with the biomarkers change to evaluated the possibility of a dose effect. Exercise dose (rides) was found to correlate significantly with an increase in BDNF (*r* = 0.50, *p* = 0.04). Delayed recall errors showed a trend toward a moderate inverse correlation with increased VEGF such that better memory performance tended to coincide with increasing VEGF (*r* = −0.42, *p* = 0.08). Exosome analyses revealed a significant positive correlation between miRNA-9 expression with improved StroopA/C performance (*r* = 0.98, *p* = 0.002).

**Table 4:** Neurobiological change (0–6M) correlates with exercise dose/effort and change in cognitive function for participants in exer-tour and exer-score: Exploratory pilot data.

**Biomarker**	**Partial correlation^a^**	**Exercise effort (rides)**	**Executive function (StroopA/C)**	**Memory delayed (ADAS errors)**
BDNF	*r*	0.50	0.20	0.21
	*p*	**0.04**	0.42	0.39
	*df*	15	16	16
CRP	*r*	0.00	0.16	−0.34
	*p*	0.99	0.53	0.16
	*df*	15	16	16
IL-6	*r*	0.03	0.15	−0.06
	*p*	0.91	0.54	0.82
	*df*	15	16	16
VEGF	*r*	−0.30	−0.05	−0.42
	*p*	0.25	0.84	0.08
	*df*	15	16	16
Exosome (miR-9_N0_Rel_U6)	*r*	−0.394	0.984	−0.815
	*p*	0.51	**0.002**	0.09
	*df*	3	3	3

a *Controlling for age and sex*.

*Bold values indicates statistically significant (p ≤ 0.05)*.

### Neuroimaging^7^

**Figure 6:**
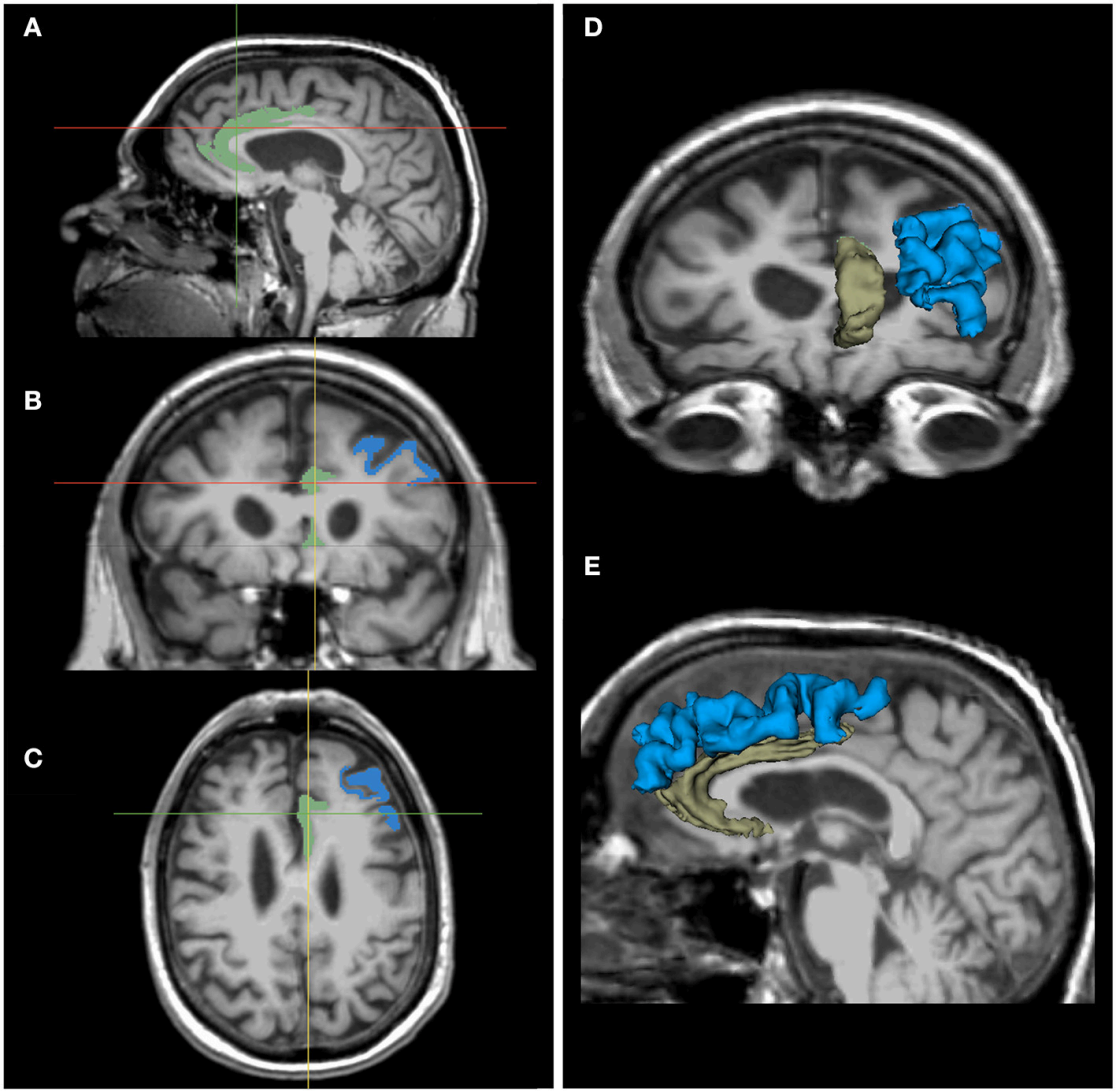
Neuroimaging correlates with exercise and cognition^13^: MRI of ACC and DLPFC. Notes: Illustration of regions-of-interest (ROI): ACC (increased with exercise dose) and DLPFC (increased with improvement in verbal memory); shown here in one individual and in one hemisphere for ease of presentation. Images of ACC (green) and DLPFC (blue) in 3 sagittal **(A)**, coronal **(B)**, and axial **(C)** images aligned by crosshairs. ROI 3D model reconstructions give a sense of their structure in 3D space, as seen from the anterior **(D)** and left **(E)** sides.

Neuroimaging (3T structural MRI) was grant-funded and sought from all participants, but not all were willing or able to undergo imaging (data from completers was available from a maximum of *n* = 8 for analysis). Partial correlations, just as described above, are reported in Table 5. Greater exercise dose (number of rides over 6M) was positively correlated with increasing PFC (*r* = 0.80, *p* = 0.003) and ACC (right; *r* = 0.70, *p* = 0.05; Figure 6). Verbal memory (delayed recall) errors were inversely related to increasing DLPFC, such that improvement in memory corresponded to increasing volume (*r* = −0.80, *p* = 0.01; Figure 6).

**Table 5:** Neuroimaging change (0–6M) correlations with exercise dose/effort and changes in cognitive function (0–6M) for participants in exer-tour and exer-score: Exploratory pilot data.

**MRI ROI**	**Partial correlation^a^**	**Exercise effort (rides)**	**Executive function (StroopA/C)**	**Memory delayed (ADAS errors)**
Hippocampus (left)	*r*	0.07	0.22	0.02
	*p*	0.86	0.55	0.95
	*df*	6	8	8
Hippocampus (right)	*r*	0.14	0.14	0.08
	*p*	0.74	0.69	0.83
	*df*	6	8	8
DLPFC	*r*	−0.10	0.19	−0.80
	*p*	0.84	0.62	**0.01**
	*df*	5	7	7
PFC	*r*	0.89	0.51	0.07
	*p*	**0.003**	0.13	0.85
	*df*	6	8	8
ACC (left)	*r*	−0.42	−0.36	−0.43
	*p*	0.30	0.30	0.21
	*df*	6	8	8
ACC (right)	*r*	0.70	−0.14	0.00
	*p*	**0.05**	0.70	1.00
	*df*	6	8	8

a *Controlling for age and sex*.

*Bold values indicates statistically significant (p ≤ 0.05)*.

## Discussion

This RCT was an attempt to replicate and extend findings of prior research, which found greater cognitive benefit for older adults engaged in interactive mental and physical exercise (i.e., while exergaming using a cybercycle; Anderson-Hanley et al., 2012) and furthermore, given pilot data which suggested greater mental challenge may increase cognitive benefit of exergaming (Barcelos et al., 2015). This RCT examined whether similar effects would be found among community-dwelling older adults with or at risk for MCI (screened as MCI; sMCI). We hypothesized that a more mentally challenging exergame would yield greater benefit to executive function than a more passive exergame. Community dwelling older adults, with a focus on those with sMCI, were randomly assigned to: exer-tour (pedaling along a virtual bike path), exer-score (pedaling to score points by navigating an interactive videogame), or a game-only condition (the latter having to be adapted due to issues with participant adherence, resulting in a shorter and remunerated, quasi-experimental arm).

Fourteen older adults with sMCI were adherent to the minimum assigned dose (average 3 × /wk of exercise) for 6 months. Both conditions were found to yield significant improvement on one of three measures of executive function (Stroop A/C), producing similar moderate effect sizes at 6 months (*d* = 0.52 and 0.47, respectively). There was no interaction effect; however, from informal examination of the graph of the data (Figure 4), it appears that the exer-score condition did not produce incremental gains at the mid-point (3M), while the exer-tour condition did appear to yield benefit sooner, although by 6 months the exer-score and exer-tour gains were similar. It is hypothesized that since the exer-score condition does require significant mental effort to play, it may have taken longer for exer-score participants (with sMCI) to master the interactive pedaling and game-play. Thus, there may have been delay in triggering the synergistic effects of the interactive exercise that would lead to a neurobiological cascade of events ultimately linked to any cognitive benefit. Furthermore, it may be different brain networks are activated by various aspects of the interactive physical and mental tasks in each condition, thus resulting in differential enhancement of network functions; for example, the effect on Stroop herein is consistent with activation of the fronto-parietal network used in inhibition and switching and the fact that the volume of the ACC as shown on MRI imaging was enlarged proportionally to exercise dose further lends support to the impact on that specific network (Grandjean et al., 2012).

The differential relative impact of the two forms of interactive physical and cognitive exercise, exer-tour and exer-score, was apparent when comparing mid-point (3M) moderate effect with small effect (*d* = 0.52 and 0.14, respectively), while the game-only condition showed little impact (i.e., of cognitive training; *d* = 0.13). By contrast, the pedal-only condition by 3M was more consistent with the literature on the benefits of physical exercise alone for cognition as it yielded a significant within-group improvement (Colcombe and Kramer, 2003), although it was a more modest effect (*d* = 0.35) than the exer-tour, which is also consistent with past research in which the cybercycle effect exceeded exercise alone (Anderson-Hanley et al., 2012). It may be that the measures of executive function that were chosen were not as sensitive to the particular impact of each of the conditions as they could be; while Stroop A/C revealed an effect, Trails 2/1 and Digit Span B/F did not, perhaps because the components of executive function were not particularly enhanced (e.g., task switching and working memory, respectively). Future research, could strategically select measures that might better detect change in selective attention or inhibitory control which arguably might be more affected by repeated use of the exer-score condition in which one is chasing dragons while scanning the horizon for additional opportunities and avoiding pitfalls, and so forth (e.g., Flanker or Go/no-Go tasks might have greater sensitivity to detect for specific and relevant changes in executive function; Diamond, 2013).

Analysis of secondary outcomes from the present study yielded promising indications that this type of exergaming activity can yield cognitive gains in other realms of cognition^12^ (e.g., verbal memory) among individuals with sMCI (with exer-tour outperforming exer-score). Moreover, these effects seem to generalize beyond standardized tests of cognition, to self-reported perceptions of everyday cognitive function and also vigor^13^ (again, with exer-tour outperforming exer-score). The latter finding may benefit from further probing, perhaps via focused exit interview questions regarding possible reasons for this curious finding. Perhaps the regular practice of a mentally challenging videogame led participants in that condition to subjectively feel less capable (even though by 6M the gains exer-score made in executive function matched that of exer-tour). It is also likely that there would be individual variability in response to the gaming scenarios (e.g., prior research has found that one's trait competitiveness can interact with benefit from presumably facilitative features of an exergame, such as the presence or absence of an avatar (Snyder et al., 2012). Indeed in conduct of this trial, anecdotally, it was noted that some individuals were keenly enthused by and motivated to keep track of their score, while others were lackadaisical in their approach to pedaling around for exercise but without much care for scoring. Indeed, research shows the salience to the individual of the storyboard of a videogame, as well as other features may affect enjoyment or persistence, and can facilitate or limit use (Wang and Goh, 2017); all relevant issues to consider when designing and planning a long-term clinical trial with a goal of ensuring a thorough dose, via consistent adherence.

These findings of significant effects with both types of exergaming: exer-tour (low mental challenge) and exer-score (high mental challenge) on executive function and memory, mirrors one of the few similar studies, by Eggenberger et al. (2015), who reported similar trends when comparing an interactive dance exergame, with dual-task walking (non-interactive), and physical exercise alone. It is interesting to note that effect sizes were larger in our study and thus reached statistical significance, while the trial above had a much larger sample, but the effects were smaller leading to marginal results, despite having exergaming conditions that were in many ways similar. Perhaps this was due to the fact that the sample herein was sMCI, specifically, while the Eggenberger study utilized normative older adults. It seems likely that general, MCI participants have already begun some cognitive decline and so they may have more room to grow, so to speak, or at least more distance from the ceiling on cognitive tests to reveal change when attempting to attenuate the anticipated downward slope of cognitive decline, and thus the effect may be more readily apparent. Future research could further evaluate the possibilities by enrolling and comparing effects of both normative and MCI participants, with care taken to avoid possible ceiling effects on baseline assessments.

Finally, possible mechanisms linking exercise to enhanced cognition via presumed improved brain health were explored with promising findings from pilot data sampled from willing participants. Biomarker changes (e.g., BDNF and exosomes) were found to correspond to improvements in cognition and dose of exercise, each consistent with prior research (e.g., BDNF has been found to promote the differentiation of new neurons and synapses, and therefore, has been proposed to be a mediator of adult neuroplasticity; Huang and Reichardt, 2001; Leßmann and Brigadski, 2009; Flöel et al., 2010; Park and Poo, 2013; Edelmann et al., 2014). Furthermore, neuroimaging revealed promising gains in key ROIs (e.g., DLPFC and ACC) that corresponded with improvements in cognition and dose of exercise, again consistent with reports in the literature (Burdette et al., 2010; Weinstein et al., 2012; Chapman et al., 2013, 2016; Hayes et al., 2013; Ehlers et al., 2017; Li et al., 2017). While we retain a cautious stance for interpreting these findings given the limitations already noted, we are heartened that changes found are largely consistent with changes in gray matter volume that have been noted in other studies of learning and physical training. Some of which have reported statistically significant changes as early as 1–2 weeks (e.g., Ceccarelli et al., 2009; Zatorre et al., 2012) and specifically regarding training-induced changes in older adults before the 6-month time point (e.g., Boyke et al., 2008).

Strengths of this RCT include a study design crafted to clarify whether cognitive benefit could be maximized with a more challenging videogame component integrated with the physical exercise. In contrast to the findings of the ACES-pilot study (Barcelos et al., 2015), which found that after just three months of exer-score resulted in greater cognitive benefit than the exer-tour. The present findings suggest that for a more impaired MCI population the benefit of exer-tour shows up by three months, but it may be necessary for participants to stick with the interactive exercise through a longer window, six months, in order to master the challenges and reap the benefits of the enhanced exercise experience. Additionally, this study design began to tease apart the impact the of component parts of exergaming (e.g., physical exercise only vs. mental exercise only), as well as explore possible neurobiological mechanisms and neuroimaging markers that tentatively shed light and nudge future research regarding the ways in which the body and mind might interact for neuroprotection or amelioration of cognitive decline. A particular strength of this study is the use of alternate forms and additional serial testing (via single bout and 3M evaluation) that preceded the final 6-month evaluation such that practice or learning effects should have largely “washed out” or likely plateaued by that point (Beglinger et al., 2005), thus greater confidence can be placed in the results as representing a genuine effect.

A significant limitation of this study is the difficulty participants experienced in engaging a new behavioral approach to preventing or curbing cognitive decline. Attrition far exceeded our expectations based on past pilots and standard rates noted in the literature. The fact that this study focused on persons with sMCI, who face more than the usual challenges in starting a new exercise regimen, was likely a major contributing factor. Additionally, the requirement set out initially by grant reviewers and medical center IRBs that persons with MCI be considered a vulnerable population and must exercise in a limited environment with professional oversight (i.e., physical therapy clinics) proved daunting as participants were unwilling or unable to arrange transport 3–7 × /wk to engage in regular exercise. Obtaining approval to make the equipment more accessible within retirement communities and neighborhood wellness centers did help somewhat, but nevertheless, retention remained a significant issue. The resultant small sample does diminish statistical power, although despite this, effects were sizeable enough to detect. Additionally, a small sample limits our ability to ask nuanced questions of the dataset which the study design would have facilitated if a larger sample had been obtained. For example, it was an a priori goal to compare known subtypes of MCI (i.e., amnestic vs. not) to see what, if any, impact subtype of MCI might have had on participants' abilities to learn and engage the more challenging exer-score videogame. This will have to wait for a future trial with a larger sample, perhaps by recruiting, as was necessary herein, those yet undiagnosed, but going beyond a screening tool, a future study could prospectively build in the mechanisms for a complete clinical workup of MCI as well. Nevertheless, much was learned regarding the needs of MCI patients and families in the conduct of such a trial, and will enhance future trial designs and interventions (see below). It will remain challenging however, to balance ideals about statistical power with the reality and importance of continuing to conduct research with clinical samples.

Future research is needed to address limitations of this study and improve retention and adherence to these types of behavioral interventions which have promising findings despite the variety of hindrances. For example, to try to address the challenges of enrolling and retaining MCI participants, our lab has been developing a more strategic and tailored in-home approach to facilitate adoption of this type of long-term behavioral intervention. We have developed a portable, adaptive, tablet-based neuro-exergame^14^, *Memory Lane*™, specifically designed to target executive function by way of integrating specific mental challenges presented via a relatively simple, in-home interactive Physical and Cognitive Exercise System (iPACES™^15^). While still only available for research purposes, the design had limited bulkiness of equipment and used budget components such that a large clinical trial will remain feasible (for example, an under-table pedaler paired with a budget tablet). Preliminary results from single bout use of our prototype iPACES™ have been promising (Anderson-Hanley et al., 2017), and we hope these various studies will encourage others to identify, design, and implement novel behavioral interventions to combat and ameliorate cognitive decline. Indeed there are a number of other open protocols currently recruiting to innovative behavioral interventions (Legault et al., 2011; Lee et al., 2016); for example, the Aerobic Exercise and Cognitive Training Trial (ACT) out of the University of Minnesota is enrolling patients with MCI to better understand the benefit of combined physical and mental activities (NCT03313895), and the Multidomain Alzheimer Prevention Trial (MAPT) combines physical and cognitive exercise, while also layering a nutritional component that may be able to further maximize any cognitive benefit derived (Vellas et al., 2014). Some research teams have been pushing the envelope on the possibilities given advancing technologies and have even explored web-connected exergaming to facilitate participation (Bamidis et al., 2011; Konstantinidis et al., 2017). The availability of such innovative interventions through data-yielding clinical trials is heartening to many individuals and families already facing the challenges of cognitive decline.

Given the encroaching dementia epidemic, it behooves researchers and society at large to continue to press for innovative and effective solutions that aim to maximize cognitive benefits. In this way we can hope to make meaningful contributions to the important cause of slowing or preventing the onset of cognitive decline. It is likely that a multi-factorial approach will yield the greatest impact, but there is much work to be done to clarify which components and intensities (e.g., of physical and mental exercise, nutritional and/or social supports, etc.), to meld and leverage for the best and most meaningful impact on brain health so as to ensure sustained cognitive functions in later life. Furthermore, it seems likely that there is a great deal to learn about factors that predict response to any intervention, including physical and mental exercise. It may be that certain genetic factors, such as ApoE status may moderate the body's ability to spark a given neurobiological cascade and thus reap the benefits of a given intervention such as interactive physical and cognitive exercise (Raichlen and Alexander, 2014). The more we learn about these underlying mechanisms and the nuanced effects exposed by analyzing larger samples, the more effective we can be in designing and tailoring interventions (Morrison-Bogorad et al., 2007; Lieberman, 2009; Larson, 2010; Read and Shortell, 2011; Gerling and Mandryk, 2014; Barha et al., 2016) to do the most good for the greatest number of people as fast as we can, especially now that so many are faced with sliding into the long suffering.

## Ethics statement

This study was carried out in accordance with the recommendations of the Institutional Review Board (AMC, SV, VA, and Union College) with written informed consent from all subjects. All subjects gave written informed consent in accordance with the Declaration of Helsinki. The protocol was approved by the IRB.

## Author notes

Thank you to:

- Our participants and site administrators from: Albany Medical Center, Stratton VA Medical Center, Sunnyview Rehabilitation Hospital, Beverwyck, CDPHP Wellness Center, Coburg Village, Glen Eddy, Hawthorne Ridge, Jewish Community Center of Niskayuna, Kimgsway Community, Prestwick Chase, Schaffer Heights, and the YMCA of Saratoga Springs.
- Research assistants: Chris Avanessian, Joel Gideon, Julie Warren, and the NY6 2016 Summer Research Team: Elizabeth Altman, Zachariah Arnold, Amelia Denney, Casey Terzian, Jennifer Vu, and Emily West.
- Colleagues who provided advice and assistance at various stages of this project: Drs. Adam Brickman, Amanda Damjanovic, Michael Hogan, Michael McCann, and Jeffrey Woods.
- Dr. Gaetano Pastena and the clinical team at Community Care Niskayuna and Latham who assisted in collection of 3T MRI imaging.
- Ross Stensrud of Interactive Fitness Holdings for technical assistance with data retrieval from the Expresso bike platforms utilized in this study.

Earlier versions of these results were presented at the annual meetings of: the Society for Neuroscience Chapter, the International Neuropsychological Society, and the American Academy for Clinical Neuropsychology.

## Author contributions

CA-H, NB, MM, VY, DH wrote pieces of the manuscript; CA-H, NB, EZ, RG, MD contributed to study design, implementation and interpretation; BC contributed to biomarker data analysis and interpretation; KM contributed to exosome study design, data analysis and interpretation; DH contributed to MRI data analysis and interpretation; PA contributed to study design, exercise science factors, implementation and interpretation; AK contributed to study design, interpretation.

### Conflict of interest statement

The authors declare that the research was conducted in the absence of any commercial or financial relationships that could be construed as a potential conflict of interest. The reviewer DM and handling Editor declared their shared affiliation.

## Footnotes

^1^Exergaming is a subset of health games, sometimes referred to as active games, that combine physical exercise with interactive gaming features, often through a virtual reality-type platform (Van Schaik et al., 2008; Read and Shortell, 2011; Stanmore et al., 2017).

^2^Screened as MCI (sMCI) based on a MoCA cut-off score for MCI of < 26 (Nasreddine et al., 2005). This screening approach was utilized after initial planned attempts to enroll clinically diagnosed patients with MCI (e.g., recruited from neurologists, neuropsychologists, and geriatricians, yielded patients that had often already progressed in their decline toward dementia). Indeed, by the time some patients had been referred for clinical evaluations, received a formal diagnosis and subsequently were referred to our study, their disease had progressed such that they no longer fit our target population of MCI. As a result, attempts to enroll such referrals led to exclusion as the patient sometimes would be unable to understand or participate in the research and/or complex intervention procedures.

^3^This difference is surmised to be likely due to differences in recruitment as the prior, Cybercycle Study, only included participants exercising at their residential facility, whereas the present study included those that could travel to exercise equipment, often at locations across town.

^4^single-bout results are not reported herein in part due to space constraints, but also given the focus of the RCT on long-term intervention results which necessarily analyzes a different/smaller sample of those adherent for 6-months; furthermore the single-bout was not a part of a priori hypotheses in the study design, but rather included to accompany the training session and provide a repeat testing experience/familiarization with tests before the follow-up evaluations (3 M and 6 M) which would partly help “wash out”/reach sooner the plateau of practice/learning effects and thus more plainly isolate intervention effects at the follow-up evaluations; finally, the single-bout assessment provides a consistent pattern with our prior RCTs (the Cybercycle Study and the ACES-pilot) in which similarly a second round of testing before the long-term outcome for similar reasons, as such this increases comparability of our data across trials and time-points (both in terms of amount of familiarization with testing and matching of alternate forms at each time-point).

^5^http://surfer.nmr.mgh.harvard.edu/

^6^total gray matter volumes were the focus of this study (white matter not considered herein)

^7^https://surfer.nmr.mgh.harvard.edu/fswiki/LongitudinalProcessing

^8^While the PFC and ACC overlap, they are reported separately here, in an attempt to replicate prior reports in the literature which identified change after exercise in each ROI.

^9^The 3M analyses were not limited to sMCI, because added comparison of the fourth group at 3M (the pedal-only condition from the archival dataset; Anderson-Hanley et al., 2012), could not be sorted by the same sMCI criteria used in the present study (MoCA scores were not available for pedal-only participants). Thus, the decision was made to utilize all adherents (normative + sMCI) at 3M from the four groups to retain comparability within the 3M comparisons.

^10^Results for bioassay and neuroimaging data analyses are to be interpreted tentatively as exploratory pilot results, given the small sample sizes even with collapsing across conditions.

^11^Additional cognitive domains that might be sensible to assess in a follow-up study, might include visuospatial skills, given the integration and regular use of visuospatial tracking in the conditions with virtual reality screens.

^12^The two mood subscales (vigor and confusion from the BMS) were probed retrospectively on suggestion of an anonymous reviewer and the ex-tour reported a significantly greater sense of vigor at 6M, than exer-score.

^13^As noted above, both ACC and PFC were analyzed in order to replicate prior research which reported each ROI correlated with outcomes (e.g., exercise dose or cognition), but because of the overlap of these ROIs only one is shown here for clarity.

^14^Neuro-exergaming (Anderson-Hanley et al., 2016, 2017), is an adaptation of existing terms and used herein to indicate the integration of neurogaming with exergaming for the express purpose of benefiting brain health and fostering improved cognitive function

^15^iPACES™ 2013-present, patent pending No. 62/140,991; BSK Ref. No.: 416P1700A
